# Impact of Seed Origin and Genetic Drift of Improved Rice Variety IR841 in Benin

**DOI:** 10.1186/s12284-023-00657-w

**Published:** 2023-10-25

**Authors:** Paulin Sedah, Lambert Gustave Djedatin, Laura Yêyinou Estelle Loko, Eben-Ezer Baba Kayodé Ewedje, Azize Orobiyi, Chalemagne Dossou Seblodo Judes Gbemavo, Joelle Toffa, Cyrille Tchakpa, Philippe Cubry, Francois Sabot

**Affiliations:** 1 Ecole Nationale Supérieure des Biosciences et Biotechnologies Appliquées (ENSBBA), Dassa-Zoumé, Bénin; 2 Ecole Normale Supérieure de l’Enseignement Technique (ENSET), Lokossa, Bénin; 3 Centre de Recherche Agricole Plantes Pérennes (CRA-PP- INRAB), Pobè, Bénin; 4 DIADE unit, UM, CIRAD IRD, Centre IRD de Montpellier, 911 Agropolis BP 604501, Montpellier Cedex 5, F- 34 394 France; 5 JEAI-GRAB, Ecole Nationale Supérieure des Biosciences et Biotechnologies Appliquées (ENSBBA), Dassa-Zoumé, Bénin

**Keywords:** Genetic drift, Seed origin, IR841, Rice Benin

## Abstract

**Background:**

Rice production is important for food security in Benin, with a national production largely dominated by the cultivation of the aromatic ecotype IR841, by far the most appreciated by Beninese consumers. However, to maintain agronomical qualities of a given cultivar, the origin and quality of seeds are mandatory and at the heart of the maintenance of yield and of market requests. Following this idea, the objective of the current study was thus to investigate the genetic purity of IR841 varieties collected from rice producers across 22 villages in Benin in relation to their agronomical performances.

**Results:**

For this, agromorphological evaluation of 72 accessions based on 13 quantitative descriptors followed by genotyping with the Illumina Infinium rice 7 K SNP array of 9 accessions was carried out in the presence of 2 controls. Agromorphologic as well as genetic and phylogenetic analyses revealed two groups, the first one Okouta97, Koum47, Nana30, Man118, Ang1 and control sample IR841-2) was characterized by seed accessions provided by the formal seed system, while the second (Koum53, Tchaka41 and Koud46) comprising seeds from local markets or from previous harvests and showing a depression in agronomic performances.

**Conclusion:**

We showed that IR841 seed purity is mandatory for the completion of agronomical performance, and that the farmers’ choice of seeds must be guided and informed to ensure sustainability and food security.

**Supplementary Information:**

The online version contains supplementary material available at 10.1186/s12284-023-00657-w.

## Background

The origin and quality of seeds are major factors for the sustainability of agronomic research, as the maintenance of yield depends on the purity of seeds (Bora et al. 2016). Rice is the staple food of more than 20% of humanity, and one of the main levers for reducing hunger and poverty in low-income countries; in Benin, it even constitutes by far the basis of food security for populations (Achigan-Dako et al. 2014; (Ndindeng et al. 2021). Rice is here consumed daily in both urban and rural areas, and represents the third cereal cultivated after maize and sorghum (FAOSTAT 2020), with the fastest growing consumption as it is the main source of energy for consumers (Codjo et al. 2021). Since the relaunch of the rice sector in Benin in 1997, paddy rice production has increased considerably from 26,981 tonnes in 1997 to reach 406,000 tonnes in 2019 (FAOSTAT 2020). However, despite these production efforts, Benin is still dependent on rice imports, with a rate of over 50% of its consumption (Tondel et al. 2020).

Indeed, rice production has increased sharply in Benin in recent years, mostly because of the diversification of rice germplasm through the introduction of several improved varieties (e.g. interspecific varieties between *Oryza glaberrima* Steud. and *Oryza sativa* L. or inter-subspecific hybrids). According to the Beninese Catalog of Plant Species in Benin (CaBEV 2), there are 19 improved rice varieties adapted to the agroecological conditions of Benin commonly cultivated (Additional file 1), including IR841(MAEP 2016), to whose we must add other varieties not taken into account but identified with local rice producers (Loko et al. 2021). Thus, a wide range of improved rice varieties have been released each recent year through AfricaRice, INRAB, NGOs, etc. for different purposes and targets: resistance to biotic or abiotic stresses, increase in yield in specific ecologies, organoleptic aspects, and so on.

Among these improved varieties, IR841 is by far the most cultivated and appreciated one among producers and consumers for the fragrant aroma of its grain (Agbobli et al. 2007); Loko et al. 2022). IR841 is an aromatic *indica* variety resulting from the cross between IR262-43-8-1 and Khao Dawk Mali (Hargrove and Cabanilla 1979), with long white grain, a yield potential of 8 tons per hectare against 4.5 tons per hectare in a peasant environment (MAEP 2016), and a short vegetative cycle suitable for both lowland rainfed crops and irrigated crops (Batamoussi et al. 2021). However, different reports (official and unofficial) exist on the disparity in productivity characteristics of IR841 and of other elite varieties existing in Benin (Batamoussi et al. 2021). Since the introduction of improved rice varieties in Benin, and in particular of the IR841 variety, little or no work has been carried out to verify the integrity of the genetic or phenotypic background of their distributed seeds over the culture cycles. Indeed, Batamoussi et al. (2021) report that non-compliance with technical itineraries by producers and the use of uncertified seeds of elite varieties for production have led to a drop in the quality of production of these varieties. Previous studies based on adoption of improved varieties and use of certified seed showed that the adoption rate of certified seed was 37% in 2017 and 22% in 2019 (Seye et al. 2017; Dossouhoui 2019).

For improved varieties, evaluating the genetic purity of the seeds and respecting the different agronomic routes are of paramount importance to maintain the potential of their expected performances. In Benin, these improved varieties are mainly maintained and supplied by certified local farmers themselves trained by national agronomic systems or specialized NGOs (Loko et al. 2021), under the coordination of the National Agricultural Research Institute of Benin (INRAB). These trainings and certified distributors normally ensure stability in terms of phenotypes (and supposedly of genotypes) of the varieties. According to Dagnoko et al. (2020) certified genetically pure seeds would improve the quality of seeds downstream on the one hand. The objective of this study is to investigate the agromorphological and genetic purity of the rice cultivar IR841, and to understand the putative underlying causes of drifts.

## Results

### Morphological Variation of IR841 Accessions

**Fig. 1:**
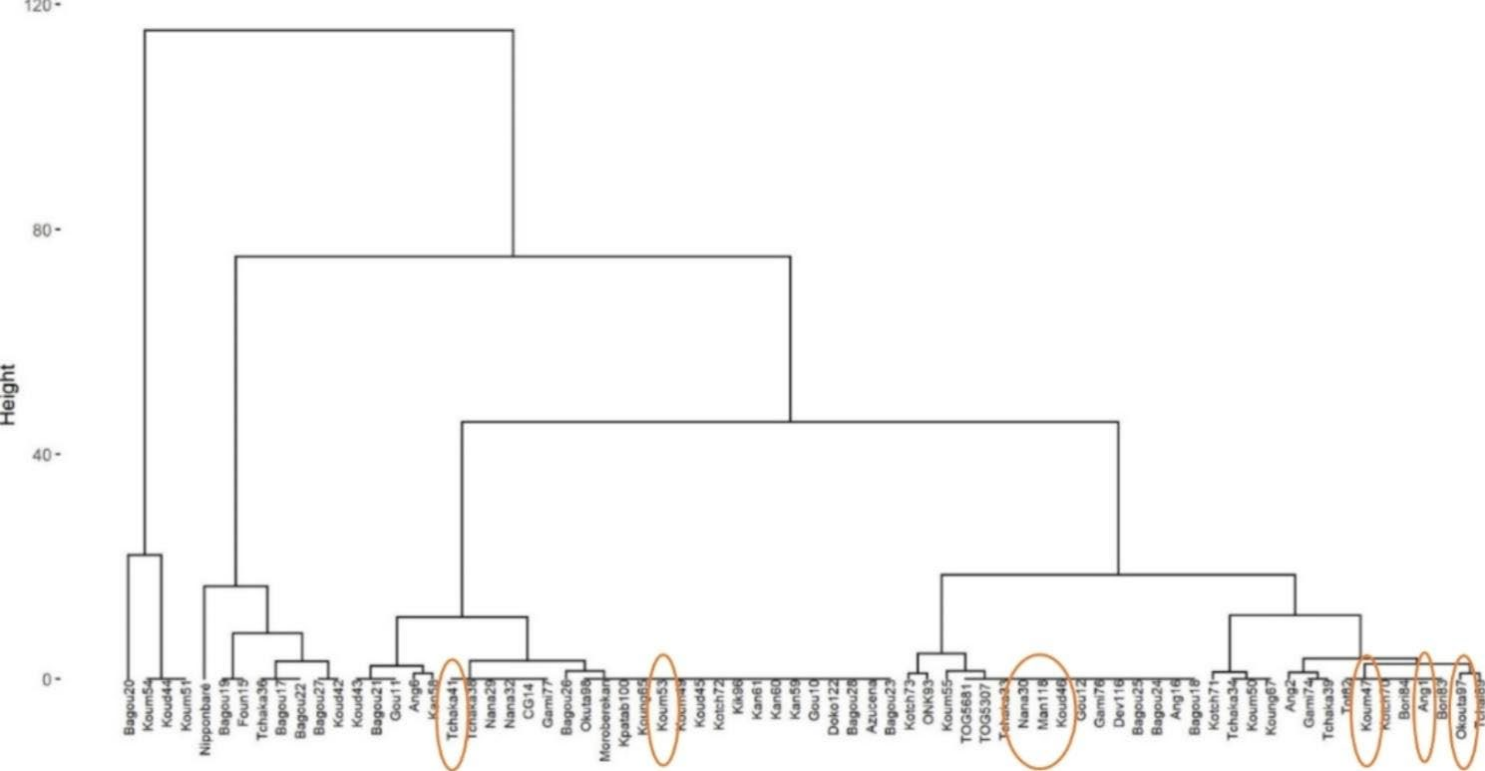
Hierarchical Ascendant Classification of the 72 accessions based the 80% of semi-heading cycle character. IR841 rice samples are boxed in color

The general Hierarchical Ascendant Classification (HAC) of all the samples shown 3 large groupings of accessions (Fig. 1), with a dispersion of the IR841 samples in a different group (Additional file 4). Compared on the results of the Principal Component Analysis (PCA) of the quantitative variables, the HAC with the characters taken individually presented the same dispersion of the IR841 accessions (Additional file 5, 6 and 7).

### Genotype and Phylogenetic Analyses

**Fig. 2:**
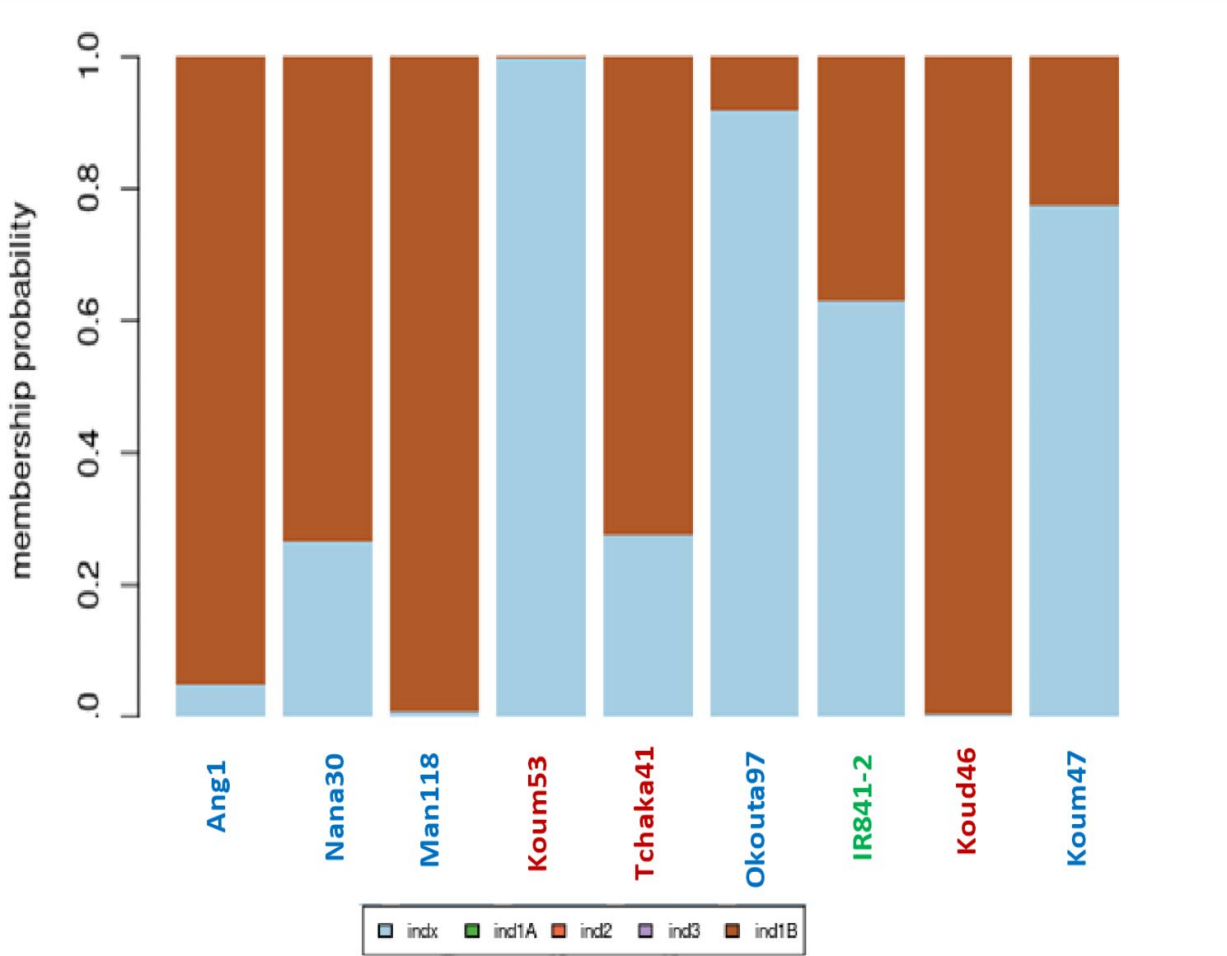
DAPC showing the variation of the genetic background within the *indica* subspecies with the IR841 accessions tested. In blue group of individuals whose seeds have been provided by approved institutions for the distribution and management of seeds. In red those obtained at the market or from previous harvests. In green the IR841 control

A total of 7,098 SNPs were generated by the 7 K SNP array chip on the IR841 rice samples. Overall, 5,527 SNPs of good quality at the threshold of 10% filtering of missing data were initially retained and after cleaning the heterozygosity at 45%, 5,170 were finally retained out of 7,098 initial SNPs. After the combined PCA analysis (see Materials & Methods), all the IR841 tested accessions fall in the *indica* subspecies group, while the Azucena control in the subspecies *japonica* and the TOG5681 outgroup in the Admix group (additional file 8), as expected. Then the Discriminant Analysis of PCA (DAPC) within *indica* subspecies showed a variation among the different genetic background for each IR841 individual, with different amount of *indx* (admixed *indica*) or *ind1B* origins (Fig. 2).

**Fig. 3:**
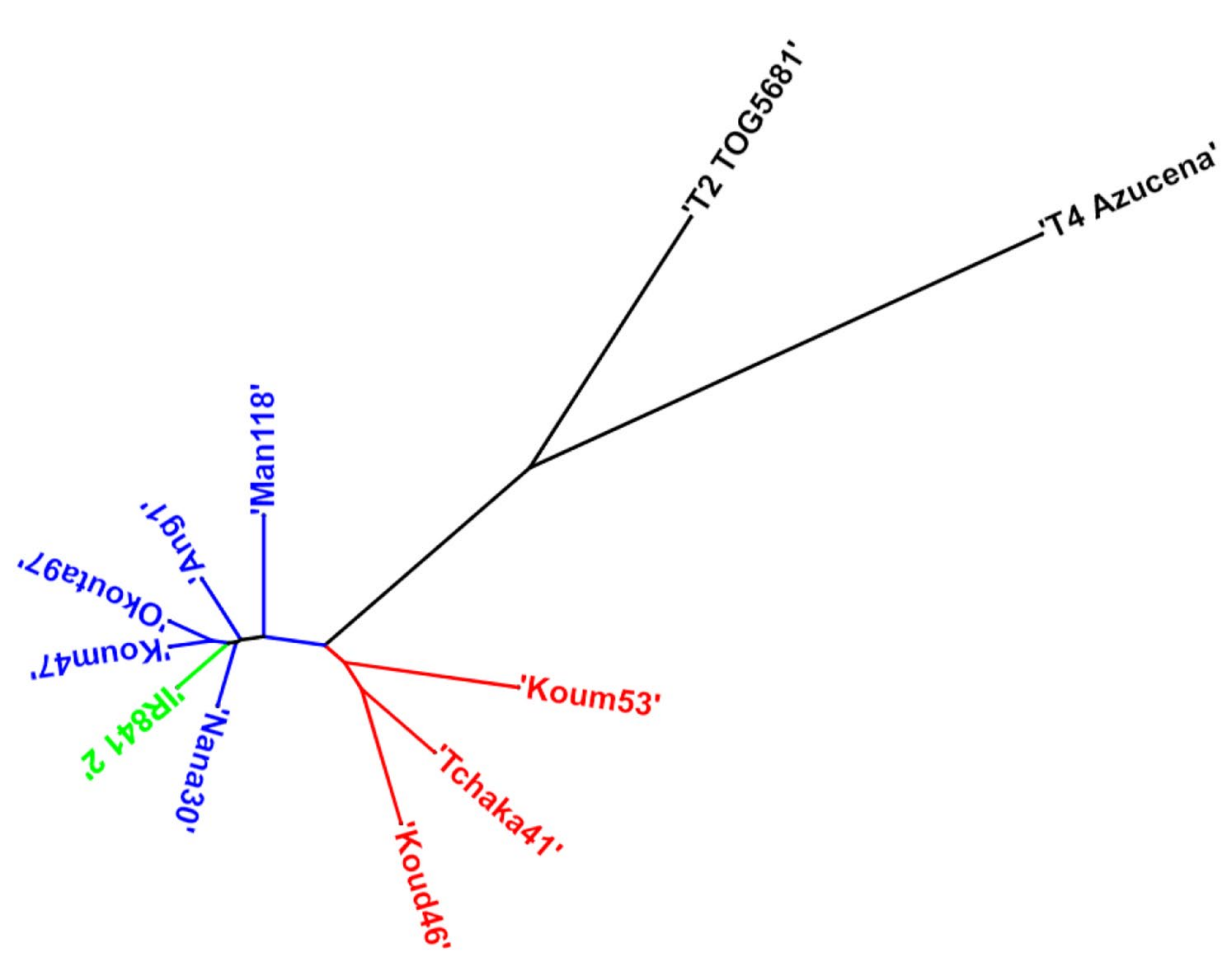
Phylogenetic tree of the 11 accessions (8 IR841 samples and 3 controls). In red, group of individuals whose seeds come from the market or from the previous harvest. In blue group of individuals whose seeds have been provided by approved institutions for the distribution and management of seeds. T2.TOG5681: *O. glaberrima* cv TOG5681 outgroup, T4.Azucena: *O. sativa* ssp. *japonica* cv Azucena control, IR841.2: *O. sativa* ssp. *indica* cv IR841 INRAB control

Based on the SNP data, the phylogenetic tree subdivided the IR841 accessions into two groups (Fig. 3), in contrast to the grouping based on the phenotyping data. The first group (Koum53, Tchaka41, and Koud46) is composed of seed for whose supply comes from local markets or from previous harvests. The second group (Okouta97, Koum47, Nana30, Man118, Ang1 and IR841-2 control sample) is characterized by accessions whose seed supply is provided by the institutions trained in the seed multiplication. The TOG5681 and Azucena controls remained separated from the two groups.

## Discussion

In different agronomic contexts, the same variety behaves differently, because the environment has an impact on the phenotypic development of cultivars with the same genetic heritage (Ullah 2011). Indeed, the richness of the soil in mineral salts and water, for example, has an effect on the size of the plants, the leaf area, the number of panicles or the weight of 1000 grains (Litardo et al. 2022). The experimental design used (Nascimento et al. 2011) minimizes or even eliminates the environmental effects, and made it possible to overcome the GxE = P interaction (Yule and Kendall 2008). Thus, the agronomic performance of improved varieties in a given environment depends mostly on the genetic purity of seeds used by farmers, and the good quality of seeds is a crucial factor that can significantly improve production up to 20% (Sahu et al. 2020); Semagn et al. 2012; Ertiro et al. 2017; Ndjiondjop et al. 2018). Thus, an improved variety (hybrid or ecotype), unlike so-called “local” non-purified varieties, should not present a variation of phenotypic and agronomic traits regardless of its geographical origin (Pusadee et al. 2009) for the experimental design allows the comparison of phenotypic data only due to the genetic basis.

However, the IR841 samples we collected from different origins, and grown in the same conditions and at the same time, presented different agronomic characteristics (1000-grain weight, semi-heading and maturity cycle, grain length, number of panicles and thallus per plants, etc.), suggesting that the phenotypic variation observed here is rather related to the genotype of the varieties tested.

Two groups of IR841 varieties were observed within the samples, one from the formal seed system and the other from the informal (market/self-harvesting) seed system. if the first group was more regular in terms of agronomic responses compared to the control line, the second group was more disparate, indicating a potential drift of these “lines” compared to the origin. By population genetic analysis, the IR841 ecotypes remained divergent with a variation in the genetic background from one sample to another as well as with the IR841 control. Similar studies have been conducted on the genetic purity of maize lines with the use of SNP markers (Ertiro et al. 2017; Josia et al. 2021), demonstrating the performance and reliability of SNP markers in genetic purity studies of cereals in general and rice in particular. The variations of IR841 ecotypes observed in the present study are probably reflection of strong admixture. Indeed, although rice is an autogamous species, this high level of diversity could be explained by gene flow between different cultivars caused by agricultural practices. For example, some growers mix seeds of different varieties for their organoleptic and culinary quality, and for their high yields (Agbo et al. 2021; Huang et al. 2022; Deng et al. 2023). Others grow different varieties together on the same field (Chen et al. 2004; Dumont 2017).

A common practice in the traditional seed farming system around the world is the sourcing of seed from previous harvests or the purchase in local markets or the exchange of seed between producers. Many cultures are subject to these practices in Africa: maize, *Zea mays* (Achigan-Dako et al. 2014), cassava, *Manihot esculenta* (Houngue et al. 2018a) yam, *Dioscorea spp*. (Aighewi et al. 2020) cowpea, *Vigna unguiculata* (Sarr et al. 2022), e.g., whose seeds are purchased at the local market or taken from previous harvests by mass selection. In other environments, it is the exchange of seed between producers either for agricultural production or for cultural purposes, for example during weddings or traditional ceremonies in the Duupa environment in northern Cameroon and in the Lokpa environment in northern Benin for the sorghum (Barnaud et al. 2008; Missihoun et al. 2012). In the latter case, seed exchange could promote popularization and adoption of the variety, but at the same time weaken the agronomic performance attributed to the variety. Indeed, through this practice, the producers, by the non-respect of the technical itineraries and the use of quality seeds (certified) of the improved varieties (as in the case of some of our IR841) for the production, gradually leads to the decline of the quality and of the yielding of the variety (Batamoussi et al. 2021).

When comparing the origin of the samples, it appeared that those belonging to the group with the control line were all from certified/trained seed suppliers. On the other hand, the second group (Koum53, Tchaka41 and Koud46) is composed of samples coming either directly from the market, or from their own harvest after having been bought at the market the previous year. Thus, their drift from the phenotypic expectation may be due to the non-purification of seeds between generations to meet these expectations. According to Sahu and Kumar. (2020), in India, a sizeable percentage of seed supply is managed by informal seed sectors, mainly by unregistered seed producers, where seed quality is questionable.

In addition, the phenotypic and genetic background variability observed within the IR841 ecotypes in this study corroborates the results obtained by Batamoussi et al. (2021) through their purification study of the IR841 variety. Their study shown through a phenotypic evaluation that only one accession out of 10 responded to the phenotypic trait of the control IR841. This once again testifies to the level of heterogeneity of the cultivars of the IR841 variety, both phenotypic and molecular, in the rice germplasm in Benin. Nevertheless, as shown in (Labeyrie 2013) the molecular and phenotypic approach to diversity does not coincide perfectly, in particular because certain local varieties distinguished by farmers do not correspond to distinct and homogeneous genetic units.

## Conclusion

In this study, we identify multiple samples from different part of Benin with the theoretically same origin/name, but falling in two different groups in term of agronomical and genetic performances. We show that the maintain of the IR841 agronomical performance can be observed only for seeds coming from trained farming systems, while the non-purification of seeds implies a decrease in performance. This highlights the need of increasing the farmer training and education on seed certification, in order to maintain the yield expectation and thus to allow hunger and poverty reduction in Benin.

## Materials and methods

### Plant Material

Rice samples were previously collected in 22 villages (Loko et al. 2021). Briefly, the local cultivars names in each village were validated through a group interview followed by an individual survey. Validation was first done in groups by rice growers using the four-square method (Kombo et al. 2012). Then, for each variety, distribution and extent, and morphological recognition traits were assessed in group while local nomenclature, abandoned varieties, reason for abandonment, varietal preference criteria, desirable and undesirable characteristics, and seed system (seed origin, seed supply constraints, seed quality, seed cost, storage method) were recorded using individual survey.

In total, 72 accessions of paddy rice cultivars collected from 22 villages in Benin were evaluated in this study (Additional file 2). Six additional varieties came from the French national research institute for sustainable development (IRD) from Montpellier, France were used as control. The study focused latter on the IR841 samples Tchaka41, Ang1, Koud46, Man118, Nana30, Koum47, Koum53 and Okouta97 and three controls samples – Azucena, TOG5681 and IR841 – as described in Table 1. The IR841 control sample was provided by INRAB.

### Agromorphological Characterization

Field experiments were carried out in the commune of Zè (Awokpa, 2°18’2’’45.888E, 6°47’8’’39.408 N), in southern Benin, belonging to the agroecological zone VI of the barren lands. (MEPN 2008). This area is characterized by a subequatorial climate with a bimodal rainfall regime (two rainy seasons and two dry seasons) and an annual rainfall ranging from 900 to 1300 mm of water (Adomou et al. 2006). The trial was conducted from March to August 2021, during the species’ growing season. During the period of the experiments, the average temperature was 27.29 °C and the precipitation index was 3.07 mm of water. The experimental system used is a random incomplete block with three replicates (Nascimento et al. 2011), and the trials were conducted in a lowland rice system, based on the technical itinerary on rice such as indicated by agronomic research in Benin, and popularized by the Territorial Agricultural Development Agency (ATDA 2019). The accessions were grown in the nursery (19 days after sowing) in 1 × 1 m plots before transplanting them in line with one plant per mound, spaced by 25 × 25 cm in between. A background spreading of manure (200 kg/ha) of chemical fertilizer NPK (15,15,15) was done during the transplanting between the pockets. A supply of 50 kg/ha of urea was carried out at the panicle initiation stage. Finally, during grain filling, a second supply of Urea (25 kg/ha) was made. Control of unwanted weeds was carried out manually in two stages. The first weeding was done at four-leaf stage, and the second after flowering. Thirteen quantitative traits and 14 qualitative traits (Additional file 3) were assessed according to the rice descriptors used by the International Rice Research Institute (IRRI 1980). Data were evaluated on 5 representative plants per accession.

### Genotyping

The INRAB reference IR841 accessions and control ones (Table 1) were pre−germinated in pots and sampled according to IRRI prescriptions. Both DNA extraction from leaves and SNP genotyping were performed at the Genotyping Services Lab from IRRI, Los Banos, Philipines, using the Illumina Infinium rice 7 K SNP array (Morales et al. 2020). The corresponding SNP data from the 3,000 Rice Genome Projet core SNP set (here referenced as the 3k core set) were retrieved from SNP−seek.

**Table 1:** List of IR841 rice samples and origin

Accession Codes	Variety names/species	Origin Accessions
Ang1	*O. sativa* ssp. *indica* IR841	Angaradébou/Kandi
Koud46	*O. sativa* ssp. *indica* IR841	Koudengou/Natitingou
Koum47	*O. sativa* ssp. *indica* IR841	Koumadogou/Boukoumbé
Koum53	*O. sativa* ssp. *indica* IR841	Koumadogou/Boukoumbé
Man118	*O. sativa* ssp. *indica* IR841	Mannonkpon/Houeyogbe
Nana30	*O. sativa* ssp. *indica* IR841	Nanagadé/Cobly
Okouta97	*O. sativa* ssp. *indica* IR841	Okouta-Ossè/Banté
Tchaka41	*O. sativa* ssp. *indica* IR841	Tchakalakou/Toucountouna
IR841.2	*O. sativa* ssp. *indica* IR841	National Agricultural Research Institute of Benin
T4.Azucena	*O. sativa* ssp. *japonica* cv Azucena	Gene bank of the Institute of Research and Development IRD-Montpellier/France
T2.TOG5681	*O. Glaberrima* cv TOG5681	Gene bank of the Institute of Research and Development IRD-Montpellier/France

### Data Analysis

First, a descriptive analysis was done with the quantitative traits to see the effectiveness of the data. Then a Hierarchical Ascending Classification (HAC) based on Principal Component Analysis (PCA) was carried out to establish the relationships between the accessions. All analyzes were done with R software v4.1.2 (R Core Team 2021). All codes and command lines are shown in Additional file 9.

All genotyping analyzes were performed according to the methodological approach described in Barro et al. (2021). First, data were filtered out of missing data up to 10% to retain good quality SNPs; a second cleaning occurred then for retaining only sites with less than 45% heterozygous sites. A principal component analysis (PCA) based on the filtered dataset and including the corresponding 3k core set was performed; the IR841 samples were superimposed on the PCA set afterward. After clustering accessions with PCA, we used the adegenet (http://adegenet.r-forge.r-project.org/) DAPC (Discriminant Analysis of PCA) package to identify discriminant functions, and then applied them to the IR841 samples to affect individuals to representative diversity clusters. For this we first run the DAPC on the identified diversity groups and evaluated the performance of the grouping. Finally, we used this discriminant analysis to assign IR841 genotypes to genetic clusters with correct precision. All analyzes were executed using R v4.1.2 software.

Phylogenetic classification was done using the distance value computed from the PCA and the Phangorn 2.8.1 (https://github.com/KlausVigo/phangorn) and Ape 5.6-2 (http://ape-package.ird.fr/) packages on R v4.1.2. Tree filtering and visualization was performed through the interactive TreeOfLife 6.5.6 (Letunic and Bork 2021).

### Electronic Supplementary Material

Below is the link to the electronic supplementary material.

Supplementary Material 1

Supplementary Material 2

## Data Availability

Dataset and figures supporting the results are included as additional files.

## Abbreviations

GxE = P: Phenotype is the interactions between genotype and environment
INRAB: National Agricultural Research Institute of Benin
DNA: deoxyribonucleic acid
NGOs: Non-Governmental Organization
NPK: Nitrogen, Phosphate and Potash (NPK fertilizers)
IRRI: International Rice Research Institute
SNP: Single Nucleotide Polymorphism
CaBEV 2: Benin Catalogue of Plant Species and Varieties 2nd edition
IRD: Institute of Research for Development
