# Supplementary material for: Impact of Seed Origin and Genetic Drift of Improved Rice Variety IR841 in Benin
Source: Rice (N Y). 2023 Oct 25;16:48. doi: 10.1186/s12284-023-00657-w (PMC10600083; doi:10.1186/s12284-023-00657-w)

**Additional files: figures**


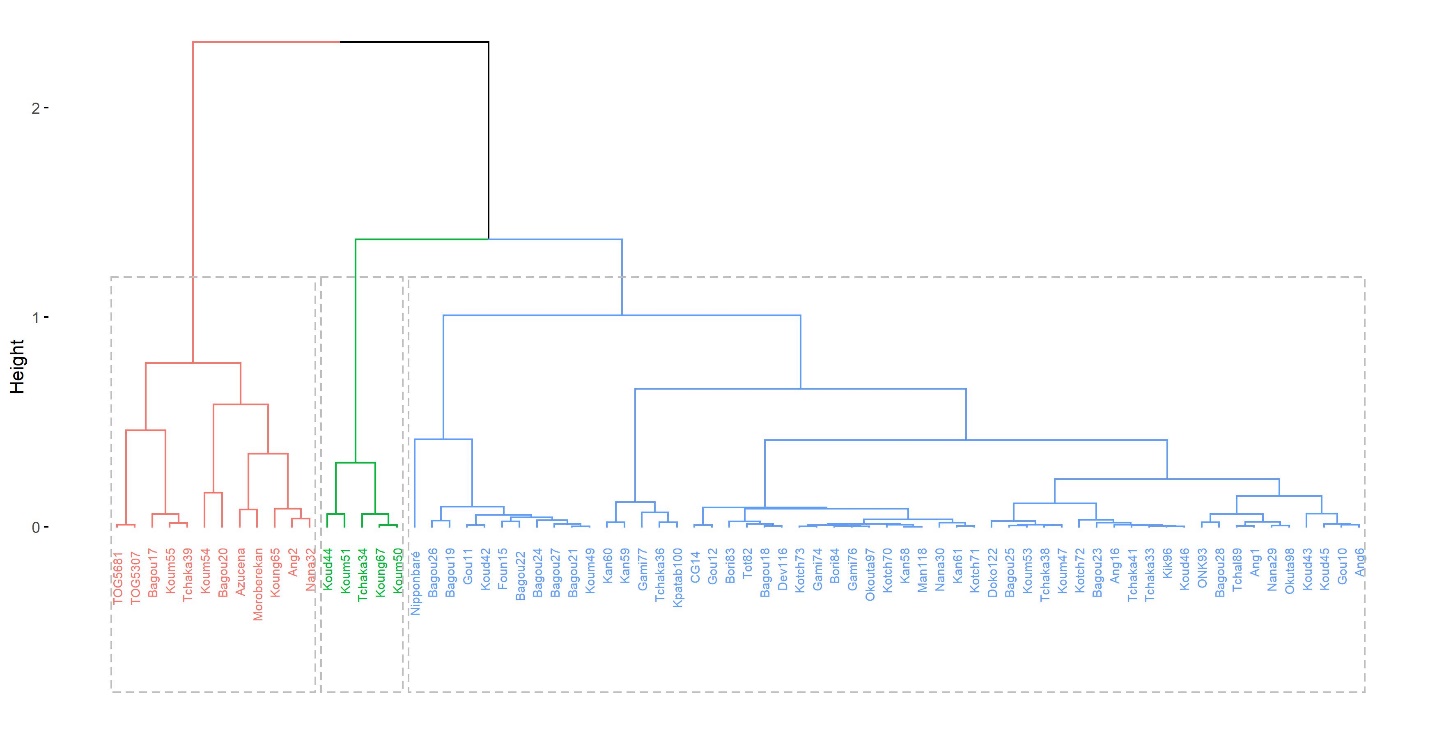


**Additional file 4** **Figure A1:** Hierarchical Classification Ascending (HAC) of the 72 accessions based all quantitative characters **
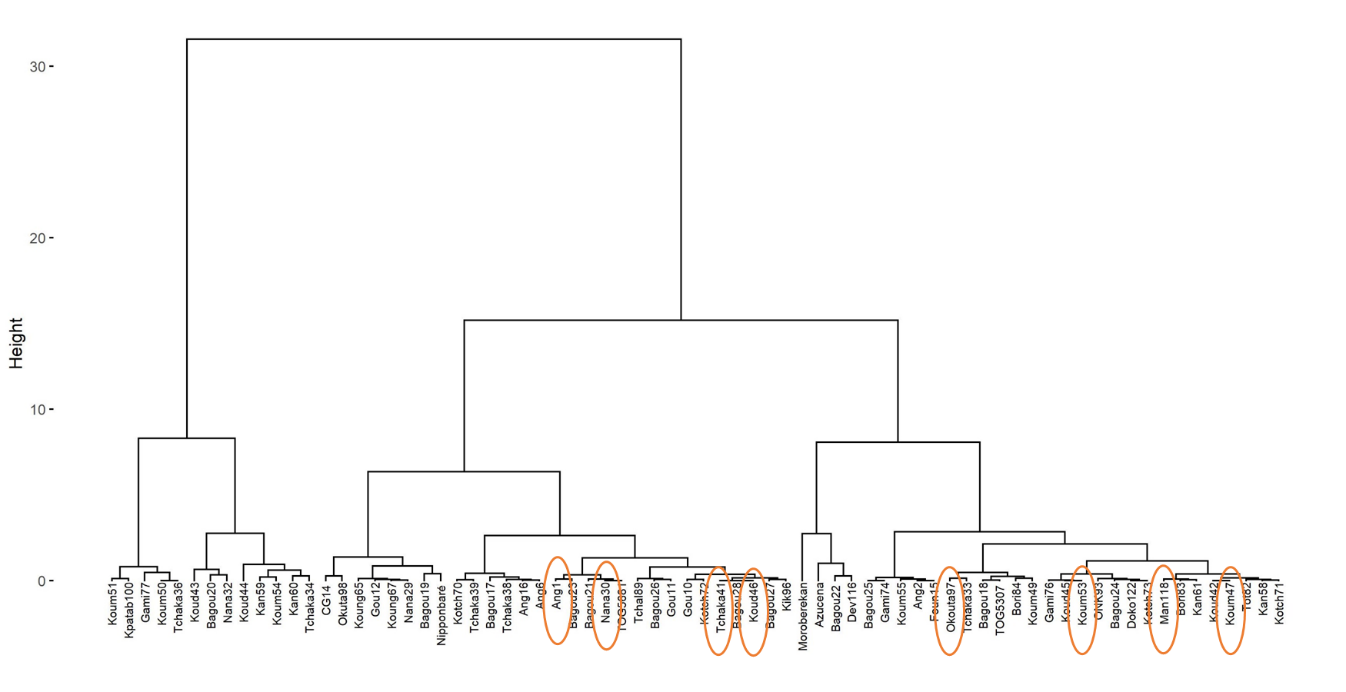
 Additional file 5 Figure A2:** Hierarchical Classification Ascending (HAC) of the 72 accessions based Number of panicles per plant characters


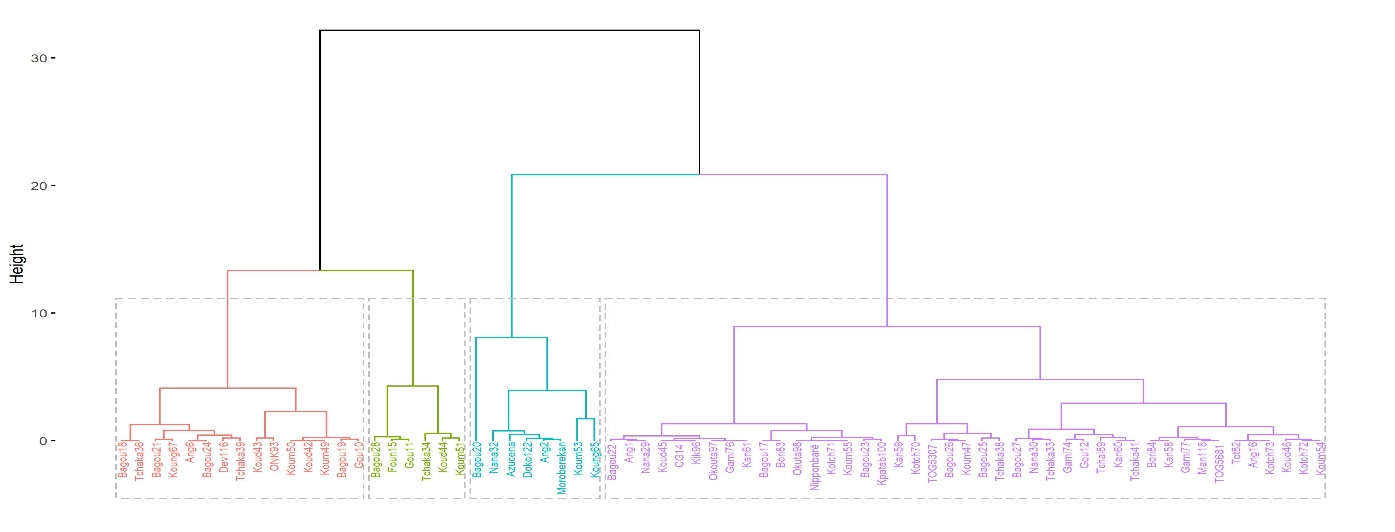


**Additional file 6 Figure A3:** Hierarchical Classification Ascending (HAC) of the 72 accessions based 1000 grain weight character

**
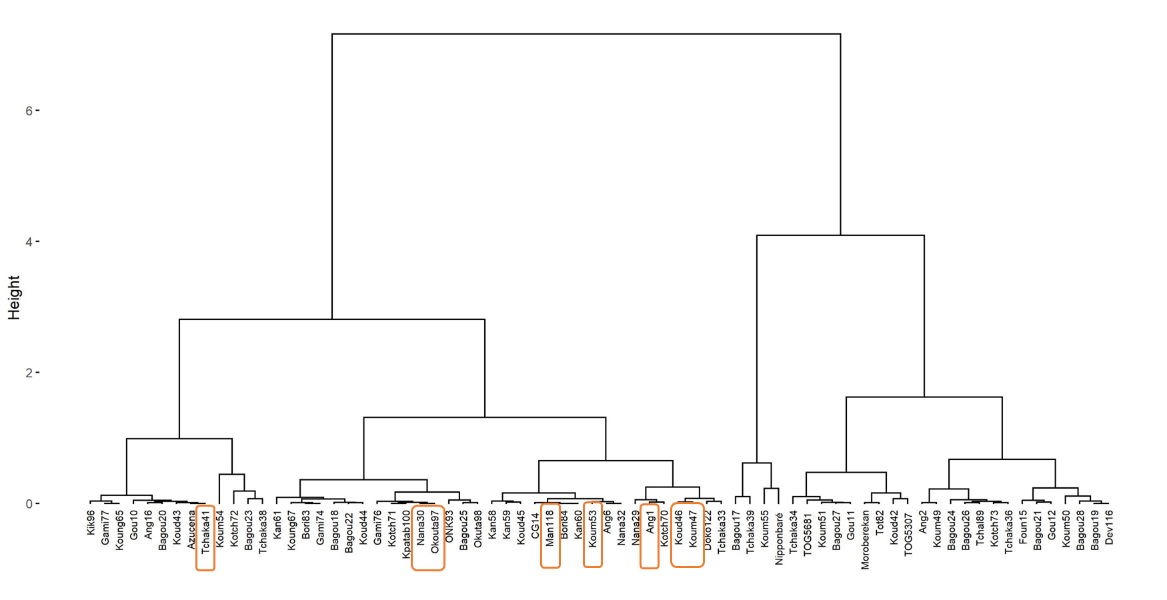
**

**Additional file 7 Figure A4:** Hierarchical Classification Ascending (HAC) of the 72 accessions based grain length character

**
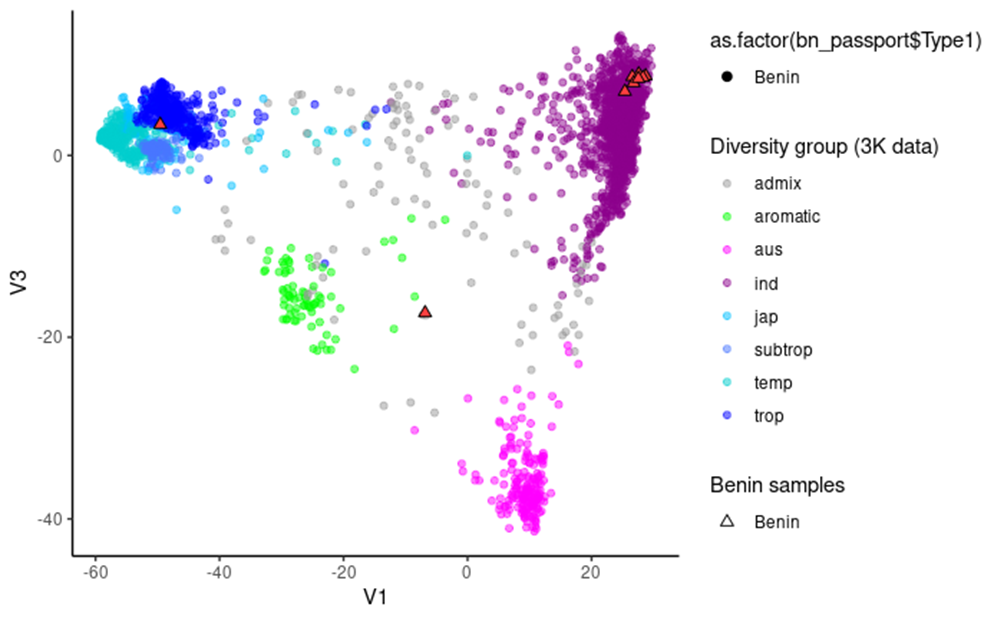
**

**
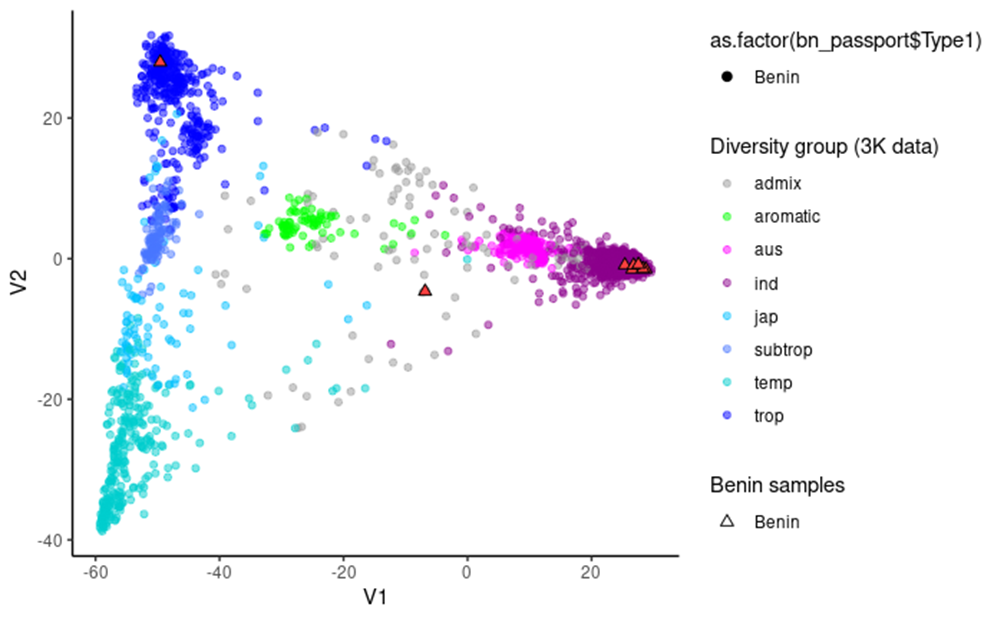
Additional file 8** **Figure A5**: Principal Components Analyses (PCA) based on SNP data for the rice reference data for worldwide diversity (3K genomes) and this study’s 09 samples from Benin and 02 from gene bank of the Institute of Research and Development/IRD-Montpellier/France


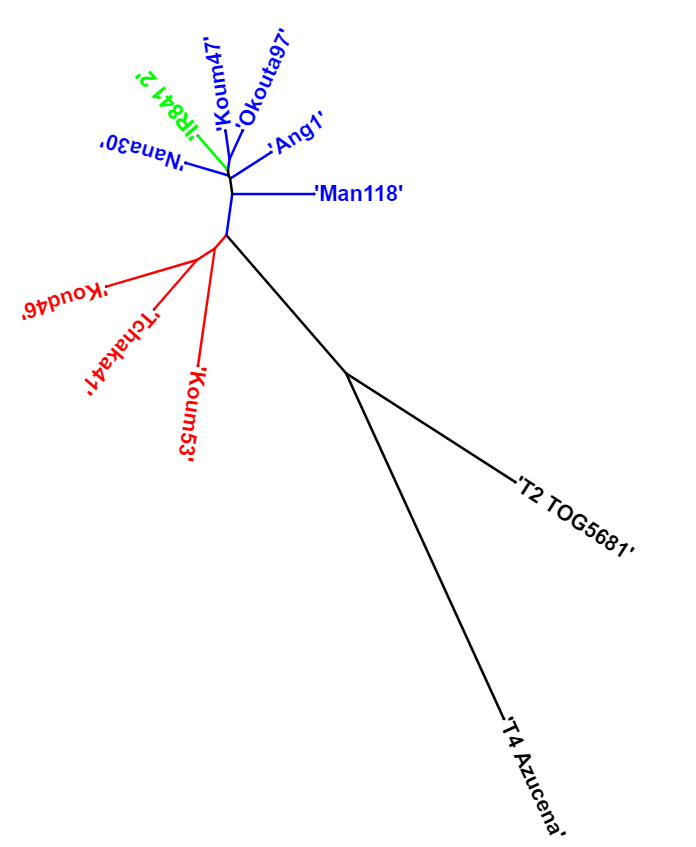

Supplement: Supplementary file 1 — Supplementary Material 1 [file 12284_2023_657_MOESM1_ESM.docx]
