# Supplementary material for: Impact of Seed Origin and Genetic Drift of Improved Rice Variety IR841 in Benin
Source: Rice (N Y). 2023 Oct 25;16:48. doi: 10.1186/s12284-023-00657-w (PMC10600083; doi:10.1186/s12284-023-00657-w)
Supplement: Supplementary file 2 — Supplementary Material 2 [file 12284_2023_657_MOESM2_ESM.docx]

**Additional files**: Tables

**Additional file 1 Table A1**: list of CaBEV 2 variety rice and some collected by Loko et al. 2021

| **N°** | **Variety names** | **Origin** | | **N°** | | **Variety names** | | **Origin** | |  |
| --- | --- | --- | --- | --- | --- | --- | --- | --- | --- | --- |
| 1 | NERICA 1 | | CaBEV2/ MAEP 2016 | | 18 | | ORYLUX 6 | | CaBEV2/ MAEP 2016 | |
| 2 | NERICA 2 | | CaBEV2/ MAEP 2016 | | 19 | | BRIZ-11 | | CaBEV2/ MAEP 2016 | |
| 3 | NERICA 4 | | CaBEV2/ MAEP 2016 | | 20 | | R8 | | Loko et al. 2021 | |
| 4 | NERICA 6 | | CaBEV2/ MAEP 2016 | | 21 | | IR15 | | Loko et al. 2021 | |
| 5 | NERICA-L14 | | CaBEV2/ MAEP 2016 | | 22 | | Pointinini | | Loko et al. 2021 | |
| 6 | NERICA-L19 | | CaBEV2/ MAEP 2016 | | 23 | | IITA3 | | Loko et al. 2021 | |
| 7 | NERICA-L20 | | CaBEV2/ MAEP 2016 | | 24 | | Gbega1 | | Loko et al. 2021 | |
| 8 | NERICA-L56 | | CaBEV2/ MAEP 2016 | | 25 | | Gbega2 | | Loko et al. 2021 | |
| 9 | IR841 | | CaBEV2/ MAEP 2016 | | 26 | | Beris 21 | | Loko et al. 2021 | |
| 10 | R4 | | CaBEV2/ MAEP 2016 | |  | |  | |  | |
| 11 | BL19 | | CaBEV2/ MAEP 2016 | |  | |  | |  | |
| 12 | BRIZ-1P | | CaBEV2/ MAEP 2016 | |  | |  | |  | |
| 13 | BRIZ-2P | | CaBEV2/ MAEP 2016 | |  | |  | |  | |
| 14 | BRIZ-3P | | CaBEV2/ MAEP 2016 | |  | |  | |  | |
| 15 | BRIZ-4P | | CaBEV2/ MAEP 2016 | |  | |  | |  | |
| 16 | BRIZ-5P | | CaBEV2/ MAEP 2016 | |  | |  | |  | |
| 17 | BRIZ-6P | | CaBEV2/ MAEP 2016 | |  | |  | |  | |

**Additional file 2 Table A2**: list of 72 rice accessions and origin. In bold the IR841 varieties used in this study

| **N^o^** | **Accessions Codes** | **Variety name** | **Village** | **Communes** | **Departements** |
| --- | --- | --- | --- | --- | --- |
| **1** | **Ang1** | **IR841** | **Angaradébou** | **Kandi** | **Alibori** |
| 2 | Ang2 | Unknown | Angaradébou | Kandi | Alibori |
| 3 | Ang6 | Wondia | Angaradébou | Kandi | Alibori |
| 4 | Ang16 | R8 | Angaradébou | Kandi | Alibori |
| 5 | Gou10 | Fondia Ibéro | Gouroubéri | Karimama | Alibori |
| 6 | Gou11 | Fondia kéno | Gouroubéri | Karimama | Alibori |
| 7 | Gou12 | Samoussagoumi | Gouroubéri | Karimama | Alibori |
| 8 | Foun15 | Dégaule | Founougo | Banikoara | Alibori |
| 9 | Bagou17 | Morri doenoun | Bagou | Gogounou | Alibori |
| 10 | Bagou18 | Essai | Bagou | Gogounou | Alibori |
| 11 | Bagou19 | Antonoumon | Bagou | Gogounou | Alibori |
| 12 | Bagou20 | Wobaga | Bagou | Gogounou | Alibori |
| 13 | Bagou21 | Yayi Boni1 | Bagou | Gogounou | Alibori |
| 14 | Bagou22 | Yoncomon | Bagou | Gogounou | Alibori |
| 15 | Bagou23 | Yayi Boni2 | Bagou | Gogounou | Alibori |
| 16 | Bagou24 | Yayi Boni3 | Bagou | Gogounou | Alibori |
| 17 | Bagou25 | R8 | Bagou | Gogounou | Alibori |
| 18 | Bagou26 | Gbéga1 | Bagou | Gogounou | Alibori |
| 19 | Bagou27 | Gbéga2 | Bagou | Gogounou | Alibori |
| 20 | Bagou28 | Yayi Boni4 | Bagou | Gogounou | Alibori |
| 21 | Nana29 | Common Kounkounga | Nanagadé | Cobly | Alibori |
| **22** | **Nana30** | **IR841** | **Nanagadé** | **Cobly** | **Atacora** |
| 23 | Nana32 | Gambiaka | Nanagadé | Cobly | Atacora |
| 24 | Tchaka33 | Bakilafema | Tchakalakou | Touncoutouna | Atacora |
| 25 | Tchaka34 | Kpantcho tèro | Tchakalakou | Touncoutouna | Atacora |
| 26 | Tchaka36 | Nérica | Tchakalakou | Touncoutouna | Atacora |
| 27 | Tchaka38 | Inaris | Tchakalakou | Touncoutouna | Atacora |
| 28 | Tchaka39 | Bakikrouma | Tchakalakou | Touncoutouna | Atacora |
| **29** | **Tchaka41** | **IR841** | **Tchakalakou** | **Touncoutouna** | **Atacora** |
| 30 | Koud42 | Nérica L19 | Koudengou | Natitingou | Atacora |
| 31 | Koud43 | Pointinini | Koudengou | Natitingou | Atacora |
| 32 | Koud44 | Timonwonti (Gambiaka rouge) | Koudengou | Natitingou | Atacora |
| 33 | Koud45 | Nérica L20 | Koudengou | Natitingou | Atacora |
| **34** | **Koud46** | **IR841** | **Koudengou** | **Natitingou** | **Atacora** |
| **35** | **Koum47** | **IR841** | **Koumadogou** | **Boukoumbé** | **Atacora** |
| 36 | Koum49 | Béris 21 (Toukounchèti) | Koumadogou | Boukoumbé | Atacora |
| 37 | Koum50 | Timonsoti | Koumadogou | Boukoumbé | Atacora |
| 38 | Koum51 | Gambiaka | Koumadogou | Boukoumbé | Atacora |
| **39** | **Koum53** | **IR841** | **Koumadogou** | **Boukoumbé** | **Atacora** |
| 40 | Koum54 | Su Itaré Kpika | Koumadogou | Boukoumbé | Atacora |
| 41 | Koum55 | Yamaboba | Koumadogou | Boukoumbé | Atacora |
| 42 | Kan58 | Moï Poua | Kankini-Séri | Matéri | Atacora |
| 43 | Kan59 | Moï Lague | Kankini-Séri | Matéri | Atacora |
| 44 | Kan60 | Moï Nihoun | Kankini-Séri | Matéri | Atacora |
| 45 | Kan61 | Moï Lague | Kankini-Séri | Matéri | Atacora |
| 46 | Koung65 | Takamorri | Koungarou | Wassa Pehonco | Atacora |
| 47 | Koung67 | Darou Morri | Koungarou | Wassa Pehonco | Atacora |
| 48 | Kotch70 | Moï Poga | Kotchessi | Tanguéta | Atacora |
| 49 | Kotch71 | Moï Touanga | Kotchessi | Tanguéta | Atacora |
| 50 | Kotch72 | Unknown | Kotchessi | Tanguéta | Atacora |
| 51 | Kotch73 | Moï Nihoun | Kotchessi | Tanguéta | Atacora |
| 52 | Gami74 | R8 | Gamia | Bembèrèkè | Borgou |
| 53 | Gami76 | Unknown | Gamia | Bembèrèkè | Borgou |
| 54 | Tot82 | IR15 | Totorou | Nikki | Borgou |
| 55 | Bori84 | R8 | Bori | N'dali | Borgou |
| 56 | Bori83 | Unknown | Bori | N'dali | Borgou |
| 57 | Tchal89 | Yayi Boni | Tchalinga | Ouaké | Donga |
| 58 | ONK93 | Toyéta | Onklou | Djougou | Donga |
| 59 | Kik96 | Nérica L20 | Kikélé-Lokpa | Bassila | Donga |
| **60** | **Okouta97** | **IR841** | **Okouta-Ossè** | **Banté** | **Collines** |
| 61 | Okouta98 | Béris21 | Okouta-Ossè | Banté | Collines |
| 62 | Kpatab100 | NERICA L56 | Kpataba | Savalou | Collines |
| 63 | Gami77 | Unknown | Gamia | Bembèrèkè | Borgou |
| 64 | Doko122 | NERICA L20 | Dokomey | Abomey-Calavi | Atlantique |
| 65 | Dev116 | NERICA L41 | Dévé | Dogbo | Couffo |
| **66** | **Man118** | **IR841** | **Manonkpon** | **Houéyogbé** | **Couffo** |
| 67 | TOG5307 | *Oryza glaberrima* | Gene Bank | IRD-Montpellier/France | France |
| 68 | TOG5686 | *Oryza glaberrima* | Gene Bank | IRD-Montpellier/France | France |
| 69 | CG14 | *Oryza glaberrima* | Gene Bank | IRD-Montpellier/France | France |
| 70 | Moroberekan | *Orya sativa* | Gene Bank | IRD-Montpellier/France | France |
| 71 | Nipponbare | *Orya sativa* | Gene Bank | IRD-Montpellier/France | France |
| 72 | Azucena | *Oryza sativa* | Gene Bank | IRD-Montpellier/France | France |

**Additional file 3 Table A3**: Quantitative characters used for morphological characterization

| **N^o^** | **Parameter** | **Codes** | **Measurement mode** | **Unit of measure** | **Measuring tools** |
| --- | --- | --- | --- | --- | --- |
| 1 | 80% Sowing-Heading Cycle | 80%CSE | Number of days between sowing and flowering of 80% of the spikelets on the panicles. | Day or (%) | Eye measurement |
| 2 | Panicle length | LongPan | Stage 7 days after flowering: average panicle length of 5 representative plants; measured from the base to the tip of the panicle. | cm | Double-decimeter ruler |
| 3 | Flag sheet length | LongFD | Stage: 7 days after anthesis: measure the length of the flag leaf, from the ligule to the tip of the blade, on five representative plants. Calculate the average to the nearest cm. | cm | Double-decimeter ruler |
| 4 | Flag sheet width | LargFD | Stage 7 days after anthesis: measure the width at the widest part of the flag leaf on five representative plants. Calculate the average to the nearest cm. | cm | Double-decimeter ruler |
| 5 | 80% maturity sowing cycle | 80%CSM | The date when 80% of the grains in the panicles are fully ripe. Number of days between actual sowing and maturity | Days or % | Eye measurement |
| 6 | Hauteur des plants | HPlt | Average height of 5 representative plants; measure from the ground to the base of the panicle | cm | Graduated board |
| 7 | Number of tillers per plant | NTal/Plt | Number of tillers per plant; rely on 5 representative plants 40 days after transplanting | Without Unit | Manually counted |
| 8 | Number of panicles per plant | NPan/Plt | Maturity stage: number of panicles per plant; rely on 5 representative plants | Without Unit | Manually counted |
| 9 | Grain length | LongG | Average length of 10 grains from 5 representative plants; measured with a Vernier caliper after harvest | mm | Caliper |
| 10 | Grain width | largG | Average width of 10 grains from 5 representative plants, measured with a Vernier caliper after harvest | mm | Caliper |
| 11 | 1000 grain weight | Pds1000 | 1000 kernel weight at 14% moisture content after steaming at 35°C for 24 hours; Scored for 5 Reps | g | Precision scale |
| 12 | Ratio (LongG / WideG) | RL/lG | Average ratio of length and width of 10 grains of 5 representative plants; Calculated after harvest | Without Unit | Manually counted |
| 13 | Grain thickness | EpaisG | Average thickness of 10 grains, measured with a Vernier caliper | mm | Caliper |
